# Non-Ionizing Radiation in Swedish Health Care—Exposure and Safety Aspects

**DOI:** 10.3390/ijerph16071186

**Published:** 2019-04-02

**Authors:** Kjell Hansson Mild, Ronnie Lundström, Jonna Wilén

**Affiliations:** Department of Radiation Sciences, Umeå University, S-90185 Umeå, Sweden; kjell.hansson.mild@radfys.umu.se (K.H.M.); ronnie.lundstrom@umu.se (R.L.)

**Keywords:** NIR, health care, exposure, safety, EMF, MRI, TMS, UV, Laser

## Abstract

The main aim of the study was to identify and describe methods using non-ionizing radiation (NIR) such as electromagnetic fields (EMF) and optical radiation in Swedish health care. By examining anticipated exposure levels and by identifying possible health hazards we also aimed to recognize knowledge gaps in the field. NIR is mainly used in health care for diagnosis and therapy. Three applications were identified where acute effects cannot be ruled out: magnetic resonance imaging (MRI), transcranial magnetic stimulation (TMS) and electrosurgery. When using optical radiation, such as class 3 and 4 lasers for therapy or surgical procedures and ultra-violet light for therapy, acute effects such as unintentional burns, photo reactions, erythema and effects on the eyes need to be avoided. There is a need for more knowledge regarding long-term effects of MRI as well as on the combination of different NIR exposures. Based on literature and after consulting staff we conclude that the health care professionals’ knowledge about the risks and safety measures should be improved and that there is a need for clear, evidence-based information from reliable sources, and it should be obvious to the user which source to address.

## 1. Introduction

Non-ionizing radiation (NIR) is used extensively in health care in applications such as for instance ultrasound imaging, laser surgery, and UV light treatments that have been used for a long time and magnetic resonance imaging (MRI) and transcranial magnetic stimulation (TMS) for depression therapy as examples of more recently introduced applications.

This document covers the use and safety of non-ionizing radiation in health care. NIR refers to electromagnetic radiation with frequencies from 0 Hz up to 1.1 THz, including ultraviolet, light, infrared, and radio waves, and mechanical waves such as ultrasound. The International Commission on Non-Ionizing Radiation Protection has recently published a statement on diagnostic devices using NIR, where they used the same definition of NIR [1]. In this report, we have excluded ultrasound, but therapeutic use of NIR is included.

Within the electromagnetic spectrum, NIR is situated below the ionizing radiation band that includes x-rays. NIR has less energy than ionizing radiation and cannot remove electrons from atoms, i.e., NIR cannot ionize (except for part of the UV band). NIR is sub-grouped into different frequency or wavelength bands. The different subgroups have different effects on the body and require different protection measures. The study was done as an assignment with the Swedish Radiation Safety Authority and the main aims of the study were:

- Identify and describe methods using non-ionizing radiation in health care.
- Examine anticipated exposure levels for each application and frequency range.
- Identify possible health hazards and identify knowledge gaps in the field.

## 2. Methods

Initially we investigated applications using NIR within Västerbotten County Council (VCC). Search terms (translated into English) were magnet stimulation, MRI, surgical diathermia, laser, light treatment, and UV. We also recruited a reference group of biomedical engineers at VCC, who have the responsibility for customer support, product service and maintenance with respect to all equipment listed in the inventory and, thus, could help us identify applications using NIR.

We have also used published literature, both peer-reviewed scientific literature and reports mainly from authorities such as the International Commission on Non-Ionizing Radiation Protection (ICNIRP), the Food and Drug Administration (FDA), Health Canada, and product datasheets from the manufacturers. Our aim was to investigate the exposure levels and to compare those with the present guidelines and our present knowledge of possible health effects. When available, we have also used treatment recommendations from the Swedish Medical Product Agency and national guidelines from The National Board of Health and Welfare. In some cases, we have added data from our own measurements of the exposure (UV, MRI and surgical diathermia). From the literature search and from personal communications with users and manufacturers we have tried to get a perspective over new or coming techniques using NIR. In this case we focused on applications, which are close to introduction or which have recently been introduced into the Swedish health care sector.

This project has primarily focused on the equipment routinely used in health care. Equipment that uses power in direct contact with the patient has been excluded, except for those that produce considerable electric or magnetic fields such as, e.g., surgical diathermy. This project has focused on the patient’s exposure, but when required, it has also examined exposure to the staff. The work has been divided into these subareas:

- Electromagnetic field (0–300 GHz) in health care including applications using static-magnetic field, low-frequency magnetic and electric fields and radiofrequency fields, e.g., for transcranial magnetic stimulation (TMS), MRI and electrosurgical devices.
- Optical radiation including: Visible light (400–780 nm) such as laser and light treatments and Ultraviolet light (100–400 nm) such as UV treatment and UV disinfection units.

For the broader picture of the use of EMF in health care in Sweden, we also established contact with two other County Councils/hospitals, chosen to represent a larger and a smaller County Council in Sweden. We have also investigated national as well as international guidelines on NIR that could be applied to patients and personnel. We have also searched for published information on safety aspects of EMF in health care from the Swedish National Board of Health and Welfare, Swedish Radiation Safety Authority and the Medical Products Agency. We have tried to briefly identify knowledge gap on safety and the use of EMF in health care mainly from published literature form authorities, but also in some cases from published scientific literature.

## 3. Electromagnetic Fields in Health Care

The survey at two counties and two university hospitals with primary health care facilities showed rather similar types of medical products independent of the hospital. However, the number of devices within each product category varied considerably. UV therapy units and neonatal phototherapy units were common as well as electrosurgery units. TMS equipment were more sparsely found. Laser equipment such as laser Doppler and surgical laser were also commonly found. MRI scanners are found in many hospitals and in Sweden there is an estimated 150 units. For more details see Wilén, et al. [2]

The questions about safety management systems were asked to biomedical engineers at the county and university hospitals. Some sites reported that the safety management works well, but that there are areas that could be improved, especially the transfer of knowledge to new colleagues. At the delivery of new equipment, a short education by the manufacturer is usual. Older staff members educate the new staff members. It is pointed out that a continued education is also needed and the manufactures could provide more education about safety issues.

Exposure to electromagnetic fields (EMF) is used in health care mainly for diagnostic and therapeutic purposes. The emitted EMF that are much stronger than normally encountered in our daily environment. This leads to exposure of the patients undergoing examination or treatment and the staff, e.g., physiotherapists, surgeons, radiologists. Electronic equipment, including medical implants, can also be affected by the EMF.

The main EMF interaction mechanism for frequencies up to 100 kHz is induction of electric currents. For frequencies between 100 kHz and 10 MHz, the effect is both induced currents and heat, and for frequencies above 10 MHz, the effect is seen only as heat.

There is an EU directive [3] which limits exposure to protect against known, immediate and negative effects in the staff and the patients. These limits are listed in the form of maximum induced electric field and the maximum heat load in the form of so-called Specific Absorption Rate (SAR, W/kg).

The first tactile effects of exposure to EMF at low frequencies are nerve and muscle spasms. At higher frequencies, the effects are not as clear—such as a feeling of heat, discomfort and a behavioral impact, much like having a mild fever. The strength of the EMF when these effects begin to occur is well known and is the basis of the current limits.

If the EMF exposure is below the limits, according to our knowledge available today, there are no risks associated with the exposure, either in the long or short term. However, research has shown that EMFs may exert an effect on biological systems below these limit values. The mechanisms by which this occur, and if these effects are hazardous to humans, are not known, see further SCENIHR [4]. The WHO’s International Agency for Research on Cancer (IARC) has classified both low-frequency magnetic fields [5] and radio frequency magnetic fields [6] as a possible carcinogen, class IIB.

The safety management for use of EMF in healthcare attracted attention in a workshop organized by ICNIRP, and a summary is given by Sienkiewicz [1].

**Figure 1:**
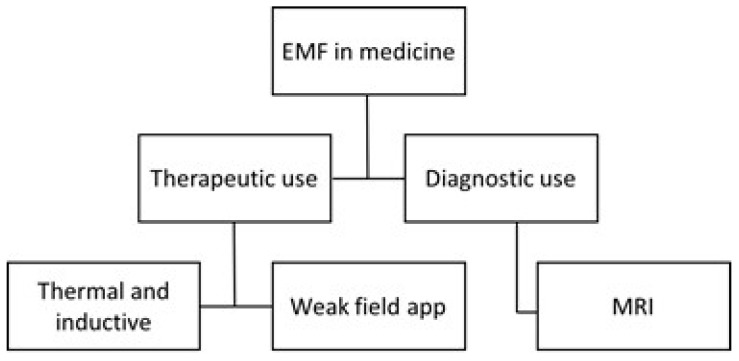
Division of electromagnetic field within the healthcare system.

The use of electromagnetic fields within the healthcare system can be divided into two main areas: therapeutic and diagnostic. This is illustrated in Figure 1. For therapeutic purposes the low-frequency magnetic fields are used to induce currents in tissues and higher frequencies are applied to produce heat. For example, induced current is used for Transcranial Magnetic stimulation (TMS), and heat development is used in diathermy, either in the form of short-wave diathermy, microwave diathermy or electrosurgery. Weak fields, with either low or high frequencies, are not used to any great extent in Sweden but are present in many places abroad. See further Markov [7] about bone and wound healing, cancer therapy, effect on microcirculation, and electroporation.

### 3.1. Magnetic Resonance Imaging

The magnetic resonance imaging (MRI) technique plays a significant role in the diagnostic portion of health care today. MRI is used for a wide range of applications, neuroimaging, cardiac imaging, musculoskeletal imaging, spectroscopy and functional imaging to mention a few. The number of areas where MRI is introduced increases and one of the recent areas where MRI has been introduced is modern radiation therapy where MRI is introduced for planning, positioning the patient, but also to monitor the treatment effect.

In Sweden today, there are an estimated 150 MR cameras and about 400,000–450,000 MR examinations are performed per year. The total number of patients may be somewhat less since some patients are getting multiple scans.

In addition to the patients, even the staff that handles the camera becomes exposed in different cases depending on the role they have in the investigation. Besides from the cameras used in the daily diagnostic there are also units in use in research and in radiation therapy where a combination of PET and MR is used. The electromagnetic fields associated with MR scanners have been studied closely [8], and have been discussed at length [9,10] and therefore only a brief summary is given here.

#### 3.1.1. Static Magnetic Fields

MR scanners in clinical use have superconducting magnets, and these usually have a cylindrical opening or bore. The static magnetic field has a flux density of 1.5–3 T. A smaller number of systems are in use in research institutions worldwide, and these use static fields up to 9.4 T. Due to the active shielding, especially for scanners with higher field strengths, the field declines rapidly with the distance from the scanner. The field is only significant within 0.5 m from the bore opening. However, this means that the static magnetic field gradient is steep, which can be of significance for the staff moving around the magnet. There is a requirement that the 0.5 mT contour around the magnet should be marked, or that access to that area is restricted. This is to prevent interference with the function of implanted pacemakers and cardioverter defibrillators. This contour is usually located inside the MRI scanner room.

So-called open systems provide far greater access to the patient and facilitate interventional procedures. In such systems, the static field is usually around 0.2–1 T.

The static magnetic field is always on and anyone who moves around the scanner will be exposed to a room-varying magnetic field, caused by movement in the static field, and its gradient, and this will induce electric currents in the body, and therefore the movement should be controlled and not too fast. These currents may lead to subjective feelings, depending on how sensitive the person is. Symptoms include dizziness, nausea, headache, and an experience of “curved” space [11,12,13,14]. 

Measurements near a 1.5 T MRI magnet revealed that static magnetic field exposure from different scanners depends on both the SMF magnet, the scanner design and the work organization [15].

ICNIRP [16] recently came with recommendations for mitigation of the induced electric field due to the movement of the static field. The value given in the ICNIRP guidelines [16] is the same as that for induced field from ELF fields, namely 1.1 V/m. This is then translated to a reference value for the time derivative, dB/dt, as 2.7 T/s.

Several studies have been reporting health complaints among the staff working in an MRI unit. Wilén and de Vocht [14] conducted a questionnaire-based study on MRI nurses, and the data indicated that 15% of nurses routinely working with MRI regularly experienced one or more adverse health complaints such as dizziness/vertigo, nausea, headaches, sleep disorders. Similar results have earlier been reported by de Vocht, et al. [17]. Schaap, et al. [18] found that people working around scanners experienced increased vertigo with increasing strength of the static field. People working in manufacturing facilities for MR scanners have been found to have a positive correlation between developments of hypertension and long-term exposure to static magnetic field [19].

#### 3.1.2. Switched Gradient Field

A switched gradient field is used for image coding, and this is emanating from three separate coils in the three directions within the scanner. Fast sequences are used to catch rapid biological events, like movement of the heart. The faster the sequence the larger time derivate of the gradient field is required. The amplitude is of the order of mT with fast rise and fall times, tens to hundreds of µs. Typically, the gradient in the region of interest may be 25–50 mT/m and the maximum value (maximum amplitude divided by the rise time, slew rate) can be 100–200 T/m/s within the picture area. The gradient field in modern systems can be as high as 100 mT/m with a slew rate of 800 T/m/s. The wave forms of the gradient field are complex and are not periodic but can be characterized by the primary frequencies in the range of kHz. For more details we refer to [2,9,10].

The limiting factor for patient exposure is the electric potential induced in the patient’s nerve fibers that can lead to peripheral nerve stimulation (PNS) [20]. To avoid this a limit of about 20 T/s—which should not be exceeded—has been given as a rheobase value. However, for shorter pulse duration higher values can be allowed [21]. 

Patients can experience slight nerve twitching on body parts that come closest to the solenoid coils, i.e., arms and legs that touch the walls of the magnet where the field is strongest. A comprehensive study on nerve excitation and changing magnetic field has been done by Reilly [20].

Occupational exposure to the gradient field may be significant, especially near the bore opening. Wilén, et al. [22] measured the rms value of the field up to 0.1 mT at 0.3 m from the center of the magnet opening. From their data dB/dt values of 70 T/s could be calculated in that position. The magnitude of the magnetic gradient field and its derivative time depend on the pulse sequence used.

Overlaid on the switched gradient signal is a ripple with a frequency around hundreds of kHz. This gives a contribution to the time derivate, dB/dt, with values up to tens of T/s. Sundström, et al. [23] measured the ripple on a Siemens Espree 1.5 T system and found values of up to 56 T/s. When equivalent measurements were done on a Philips 3 T system, the ripple was negligible.

#### 3.1.3. Radiofrequency Magnetic Fields

An RF magnetic field is usually created with a body coil integrated into the scanner, which produces a circularly polarized magnetic field called B_1_. For systems with cylindrical openings with 1.5 or 3 T, it is usually a coil in the form of a “bird cage” to provide an area around isocentre of the scanner where the B_1_-field is spatially homogeneous. Only the magnetic field component is required for the MRI, and the electric field, E, is generally low except in the vicinity of the coil windings. Employees who perform interventional procedures, especially in open scanners, where their hands and arms, and possibly head, can be exposed to levels that are like those to which the patients are exposed. The RF field has a frequency of about 42 MHz/T, meaning that for a 3-T scanner the frequency is approximately 126 MHz.

Various RF-pulse sequences are used depending on what contrast is required, and thus, the SAR value of each pulse sequence, is different. Usually, during clinical scanning, many different sequences are used to get the appropriate information. Based on our own measurements the peak values for the RF B_1_ can reach 10 A/m and more. Since the duty cycle can be about 1%, the SAR values in pulses can be rather high, in comparison with the limit values. With a whole-body average SAR value around 1 W/kg, the peak of the pulse can reach 100 W/kg.

#### 3.1.4. Limits and Regulations

There are limits and rules for the exposure of patients as well as staff who may be exposed during an MRI examination. For the patient’s safety it is necessary to comply with the standard [21]. Its adoption was intended to ensure the safety of patients undergoing an MRI examination as well as the professionals who work with MR. The standard sets both technical requirements on the equipment and on the organization of the work with MR.

The standard [21] also defines operating modes for MR systems: *normal operating mode*, *first level of control* and *second level of control* based on level of SAR and dB/dt. In *normal operating mode* the apparatus shall be set so that it does not exceed 80% of the threshold value of the PNS, and in a *first level-controlled mode* it can go up to 100%. To go to this setting requires a specific action from the operator in order to clarify that the patient or operator now is entering a special risk position.

In the EU directive [3] an exception is made for MR work so that exposure can exceed the limit values if the exposure occurs in connection with the installation, testing, operation, development and maintenance of, or research on, equipment for MRI for patients in healthcare. The exemption only applies to—some parts in the provision, and the other sections should be followed, and this applies in particular to the requirement that the employer must inform and educate the staff about the risks which can occur and how to work safely in order to avoid these [24].

For certain types of surveys, for example, when rapid processes are to be studied, it may happen that the patient can feel gentle nerve twitches in superficial nerves, which are caused by current induced in the body from the gradient field. Heating effects can occur when the number of RF pulses per unit time is high, such as in the anatomical image where detail resolution is important. 

The risk for burn injuries is especially great if the person comes too close to the walls of the magnet, since the RF field will increase with distance to the coil. Therefore, it is recommended that an insulation of at least 2-cm thickness is placed near the wall to prevent burns. At high SAR levels it is also important not to form closed loops with body parts, such as the arms over the head and the hands or knees against each other. Special consideration must be given to patients with poor body control, such as babies who do not sweat, and others with impaired circulation.

Reddig, et al. [25] have given a list of estimated absorbed energy during scanning with different MR protocols. The mean whole-body SAR value varies from 0.3 W/kg in a cardiac scan with a contrast agent to 2.6 W/kg for a lumbar spine scan. Including the time of the scan the energy absorbed ranges from 182 J/kg to 2818 J/kg.

#### 3.1.5. Risks and Effects of MRI Exposure

An MRI examination is not entirely risk-free. Because very strong magnetic fields are used, from static up to radio frequency, there are risks that require attention both before and during the investigation.

The most pronounced risk is the projectile risk. The static magnetic field is so strong that ferromagnetic objects can be dragged into the magnet with such great force that damage to the people who happen to be in the way can be life-threatening. It is important that all people entering the examination room such as staff, relatives and patients are controlled carefully so that no ferromagnetic objects are brought into the room. Incidents have occurred when, e.g., trolleys, IV stands and office chairs, have been affected by the magnetic field, attracted to and stuck to the magnet [26].

Passive implants, such as orthopedic implants, can lead to a concentration of the radio frequency field and thus provide a very local harmful warming, something that is not detected until it is too late because the heat sensors in the body are superficial. For active implants, such as pacemakers and defibrillators, the risks are that they can concentrate the radio frequency field, but also that its function may be affected. For the whole area around the implants, it may be helpful to have access to a medical specialist who has a good overview of the area and can provide advice in cases of doubt. All patients are reviewed for contraindications prior to MRI scanning where possible implants are investigated carefully. All implants are categorized as *MR-safe*, *MR-Conditional* or *MR-Unsafe.* This information is available through the manufacturer of the implants but it is a hard and time-consuming task to investigate this for each patient. Therefore, many hospitals have developed their own implant data-bases where also routines for especially MR conditional implants are stated. To keep these data bases updated is an important task for the safety organization and might be especially challenging for smaller hospitals with few MR physicists. It is imperative that the first contact with the patient for an MRI examination includes a questionnaire on which implants the patient has, and this should then be followed up before the examination. This can cause problems with people who do not remember what implants they have and when the operation history is missing. It is also important that also personnel who will be present in the MR room have been screened for various implants.

The most common problem that occurs due to the gradient field is the level of noise caused by vibrations in the wires in the gradient coils. It is a requirement that personnel in the room during scanning have hearing protection, and patients must be provided with protection. Sometimes a double hearing protection is put on the patient. Audio levels can range from 85 to 95 dBA.

When scanning pregnant account must be taken of RF heating and noise level for the uterus, but there are currently no established long-term effects of in utero MR scans. In the U.K. it is recommended that pregnant staff should avoid being in the room during scanning and that one should select sequences that minimize RF noise. This is also done in common practice at Swedish hospitals, but there exist no guidelines for this from Swedish authorities.

Some studies have demonstrated genotoxic effects in cells after exposure to an MR scan [27,28,29], while others could not demonstrate any effects [30,31]. The MRI sequences used in those studies are clinically available and routinely used in heart and brain scans. In Reddig, et al. [25], patients undergoing clinical CT scans were used as positive controls, and the authors found nearly a doubling of DNA double strand breaks 5–30 min after the CT scan as compared with before the CT scan. There was no evidence of DNA damage after the MRI examinations.

In a recent paper by Foster, et al. [32] those studies were criticized on the grounds that many lacked a positive control, sham exposure and blinding in the analysis work. They suggest that the results should be confirmed with studies with the same end points but with higher statistical power and more rigorous design.

In recent studies from Utrecht University [19,33,34] risk assessments for MRI workers have been carried out. They found an association between MRI-related occupational SMF exposure and an increased risk of accidents leading to injury, and for commute-related (near) accidents during the commute from home to work found that radiographers using intrauterine devices (IUDs) and were occupationally exposed to stray fields from MRI scanners reported abnormal uterine bleeding more often than their co-workers without an IUD, or non-exposed co-workers with an IUD. In particular, radiographers present inside the scanner room during image acquisition showed an increased risk. These findings point to the need for further research to find out if staff working close to MRI scanners are at increased health risk.

Hand, et al. [35] give recommendations on how to allocate the responsibility for safely working with MR are given. The organizations behind the document is European Federation of Organization of Medical Physics (EFOMP) and they recommend the introduction of a number of positions for the operative responsibility.

Overall there is a need for education and training of the personnel involved in MRI investigations. This should be organized in such a way that new staff members can easily get the training and information about the safety aspects. Implementing the safety management system suggested by [35] might be one way to go.

### 3.2. Electrosurgery

Electrosurgical units (ESUs) are the most common medical equipment in hospitals using EMF. RF energy is used in several surgical procedures, not only in medical care but also in dental care. In most cases, a small active electrode applicator and a flat electrode (also known as the ground electrode or return pad) are used. The ground electrode returns to the generator (monopolar configuration). The active electrode provides a high current density and serves as a cutting or coagulating instrument by applying a current with sinusoidal or pulsed waveform, and the frequencies are about 0.3 to 5 MHz. A widely used minimally invasive electro surgical procedure is radiofrequency ablation, which is routinely used in oncology, cardiology and otorhinolaryngology.

Close to the equipment the fields were quite high, but at a distance of 0.5 m from the machine, the electrical field strength fell to 32–57 V/m and the magnetic field strength fell to 0.2–0.8 A/m [36]. In the worst case at maximum reading, the surgeon’s hands were exposed to RF field with the magnetic field strength of 0.75 A/m and the electric field strength at 400 V/m. Significantly higher values were measured by Liljestrand, et al. [37] and they also noted that the fields are produced not only by the active electrode but also of the return pad and cable.

Measured peak values of the voltage at electrode tips are between 1 to 4 kV. The highest values are obtained when using coagulating mode. Wilén [38] concludes by noting that when using ESU, both surgeons and other professionals that come close to the active cable are exposed to radiofrequency fields in excess of the guidelines specified by the ICNIRP [39]. They point out that further studies and calculations must be made to ensure that the ICNIRP specified “basic restrictions” are not exceeded.

Laboratory tests show that shielding of the active conductor gives a substantial reduction of the electric field around the conductor and can thus be a possible way to move forward to reduce exposure using ESU. According to one of the manufacturers of diathermy equipment, there is one system on the market that has shielding, but the users think it is too clumsy to use and it lacks smoke exhaust. Therefore, on the Swedish market only non-shielded electrodes are used.

#### Indirect Effects of Surgical Diathermy

The use of surgical diathermy can be risky for both the personnel and patients. When the high frequency current flows in one part of the patient’s body, there is a risk of burns. The greatest risk occurs if the return current is spread over a too small area, due to poor contact with the neutral electrode. It may also be other metal surfaces the patient is in contact with which can take over a part of the return current.

The risk of current concentration during surgery in the vicinity of electrically conductive orthotics (surgically implanted metal objects such as metallic prostheses) and pacemakers is particularly important. The risk for burns increases with increasing current and power during the surgery. The lowest possible setting must therefore be used. If the patient has an active implant, such as pacemaker, bipolar cautery should be used first. Pacemaker or pacemaker electrodes must not, however, be between the surgical site and the neutral electrode. If the patient has an implantable defibrillator (ICD) the defibrillation function should be switched off during the operation.

There are also risks for explosion and fire. When the diathermy generator is used, sparks may occur. Therefore, flammable aesthetic gases should not be used, and flammable liquids, such as rubbing alcohol which could accumulate, e.g., in the navel, must have evaporated before the diathermy apparatus is used. Even the risk of ignition of bodily gases should be taken into account, especially at the opening of a dilated gas-filled colon.

The knowledge about the different settings on diathermy equipment is quite low among surgeons. The education of new surgeons is usually done on a word of mouth basis, i.e., the older ones tell the younger ones how to do it. The knowledge about the EMF emission from the equipment is very rudimentary, and precautionary measures are seldom applied, such as avoiding to place the cable close to the body or medical equipment.

### 3.3. Short-Wave and Microwave Diathermy

Diathermy is used in physical therapy for the treatment of acute or chronic orthopedic and inflammatory conditions. The therapeutic effect comes from the heat produced in the tissues due to the absorption of electromagnetic energy at high frequencies. Short-wave diathermy uses 13.56 MHz or 27.12 MHz in a continuous form or pulsed mode. Microwave diathermy uses primarily 2.45 GHz, but there are devices that work at 434 MHz. Studies for the evaluation of exposure to diathermy has mainly focused on occupational exposure by physical therapists, and we do not know of any study of patients who had undergone the diathermy. During the treatment a temperature rise is wanted, so clearly the SAR values will be dozens of W/kg during the treatment period, which may be up to 30 min.

A measurement of 20 physical therapy departments throughout the U.K. and 36 different diathermy units showed that at the distance of 0.15–0.2 m the electrical field strength for a continuous wave was generally over 500 V/m and sometimes as high as 5000 V/m when using capacitive electrodes. The magnetic field strength at the same distances was 0.5–2.0 A/m [40], and the authors suggested that the operator should maintain a distance of at least 1 m from the unit, cables and electrodes. These measurements are completely in line with what we found already in the 70s, and the devices have not changed much since then [41].

A survey of 10 short-wave diathermy devices that operated at 27.12 MHz, noted, however, that the field fell below the reference level for occupational exposure in the ICNIRP guidelines [42] at 2 m for capacitive electrodes and at 1 m for inductive equipment [43] For microwave diathermy, the measurements on about 11 units have shown that if the operator is at a 1-m distance from 2.45 GHz and 434 MHz applicators, and if there are no large metal objects in the vicinity that can reflect radiation, then the fields are within the reference levels for occupational exposure [44].

The use of this form of EMF therapy is very limited in Sweden today, and has been replaced by ultrasound.

### 3.4. Transcranial Magnetic Stimulation

Transcranial magnetic stimulation (TMS) is used both as a diagnostic instrument and for therapy. However, it is not yet widespread in Sweden, and today TMS equipment is available only at some psychiatric clinics for the treatment of depression and at clinical neurophysiology departments where TMS is used for diagnosis of nerve damage.

TMS is a technology based on the induction of an electric field inside the brain by application of an external magnetic field with rapid rise and fall times. The induced electric field in the brain has been calculated when different coils are used for the treatment. Mai and Ueno [45] reported E fields of the order of tens to hundreds of volts per meter and the induced current density was estimated at tens of A/m^2^. In this case an excitation is wanted, but it should be compared with the limits given for occupational exposure [3] which is 1.1 V/m.

The exposure of the operator during a TMS treatment session exceeded the EU directive reference levels for magnetic fields at a distance of approx. 0.7 m from the coil [46]. Given the very strong pulsed magnetic fields used in the TMS treatment, there is a clear need for safety procedures and recommendations for management. Training requirements for the staff who manage the TMS apparatus are also important.

There is a need to protect personnel against unwanted exposure, and TMS treatment is subject to the EU-regulations on exposure to electromagnetic fields [3]. The regulation has far-reaching requirements on training and information for those who may be exposed at the level of the limits, and this includes TMS treatment. We will return later to this issue in our summary with recommendations.

The Food and Drug Administration (FDA) in the United States has released recommendations for what information the manufacturer shall provide. We do not have similar requirements in Sweden. Our experience after having visited a number of institutions, which provide TMS treatment, is that the knowledge of what a TMS apparatus actually does and potential risks with magnetic field exposure is very limited.

The patient receives a high exposure that is intended to lead to nerve excitation. It is therefore necessary that the personnel operating the apparatus is well acquainted with how the TMS functions, and that particular attention must be given to certain groups of patients. Also, patients with implants are in need of special attention before a possible treatment.

Use of TMS in healthcare is not widespread. Yet. For diagnostics it is used in neurophysiological investigations, but the number of investigations is not great. From personal information from the staff at VCC it was estimated that 50–100 patients per year are investigated with the use of TMS. In psychiatry, clinical treatment with the use of TMS is only done in two places in Sweden, Skellefteå and Eksjö. The former had over 600 patients in the last half year undergoing treatment for depression. In other places, such as Uppsala, Linköping, Lund, Gothenburg, and Stockholm, treatment with TMS is used in research of this treatment.

The Swedish Medical Products Agency states in a background document [47] that today there is only moderate scientific support for use of TMS for depression. This view is shared by van Belkum et al. [48], and in their review article on the treatment of depression with TMS they write: The mode of action of this new technique is however largely unknown.

A group of European experts [49] established guidelines for therapeutic use on repetitive TMS regarding pain, movement disorders, stroke, amyotrophic lateral sclerosis, multiple sclerosis, epilepsy, consciousness disorders, tinnitus, depression, anxiety disorders, obsessive-compulsive disorder, schizophrenia, craving/addiction, and conversion. They found a sufficient body of evidence to accept with level A (definite efficacy) the analgesic effect of high-frequency (HF) rTMS to the primary motor cortex (M1) contralateral to the pain and the antidepressant effect of HF-rTMS to the left dorsolateral prefrontal cortex (DLPFC). They also stated that professionals carrying out rTMS protocols should undergo rigorous training to ensure the quality of the technical realization, guarantee the proper care of the patients, and maximize the chances of success. Finally, they predict that under these conditions, the therapeutic use of rTMS should be able to develop or increase in the coming years.

The National Board of Health and Welfare has issued a referral edition about the use of repetitive TMS as an alternative treatment for depression [50]. The method is new, but it has been used with positive effects on persons with medium to severe depression. The side effects have been light. This may lead to an increase in the application of TMS to treat depression.

### 3.5. General Comments on the Use of EMF in Health Care

The highest exposure to EMF in health care can occur in connection with the use of MR, TMS and ESU. In all three cases patients are exposed to such high levels that acute effects are possible. Also, staff can be exposed in excess of the existing exposure limits, and actions are required to ensure the safety of the staff working with these kinds of equipment.

For the patients undergoing examination or treatment, there is also a risk in connection with the use of the above techniques. Among those at particular risk are patients with implanted medical implants, e.g., pacemaker or defibrillator, or passive devices, e.g., metallic knees or hips. The knowledge of which implants can be regarded as *MRI safe* is not widespread. It would be valuable if this database could be transferred into a national database available for the staff at all MRI units in Sweden.

The knowledge of safety aspects of EMF among personnel in Sweden has not been studies or described, but the new rules from the Swedish Work Environment Authority do demand education of staff of this. A study from New Zealand on MRI technologists highlighted inconsistencies in safety practices regarding intra-orbital metallic foreign body [51]. In a study from Ghana [52] among MRI staff at a teaching hospital revealed a lack of fundamental knowledge about safety issues such as restrictions on entering the scanner room, screening patients and staff for ferrous materials before entering the room, etc. The authors concluded that their study revealed a huge training gap in the use of MRI equipment.

The latest SCENIHR report [4] recommends long-term prospective and retrospective cohort studies of personnel exposed to high-gradient field in the operation of MRI units. The DNA damage in patients after an MRI scan should be further investigated. A study on the effects of MRI exposure in children is also on the list from SCENIHR. For TMS the effect of using this type of treatment for depressions needs further studies. SCENIHR also recommends studies investigating possible cognitive effects of exposure to magnetic gradient field of employees working in the immediate vicinity of the MRI equipment.

Overall there is a need for education and training of the personnel involved in MRI investigations. This should be organized in such a way that new staff members can easily get the training and information about the safety aspects. Implementing the safety management system suggested by [35] might be one way to go.

There are quite many studies published lately on the effect of pulsed magnetic field (PEMF) on various diseases such as carpal tunnel syndrome [53], rotator cuff disease [54], and knee osteoarthritis [55]. PEMF has also been used to stimulate bone healing for non-union fractures. However, to our knowledge PEMF is not used in ordinary health care in Sweden, but we cannot rule out if it is applied in some research projects.

## 4. Optical Radiation

Optical radiation has been used in health care for different purposes for a long time. Light treatments of neonatal, dental curing light to harden composite materials are some examples. In dermatology, lasers are used for treatment of tumors and tattoos and for hair and birthmark removal. In ophthalmology, lasers are for instance used to reattach the retina, to reshape the cornea and for treatment of macular degeneration. Lasers are also found within cosmetics but these applications are not within the scope of this survey. The number of laser-based applications is steadily growing in medicine leading to an increasing number of patients and health care professionals exposed to laser radiation. Ultraviolet light is used in health care primarily to treat skin diseases such as psoriasis and eczema but also for sterilization of laboratory equipment. For the UV treatments, narrow band UVB with a wavelength peak at 315 nm or a broadband UVA light is used. Sometimes also a combination of UVA and UVB treatments are given. In UV sterilization procedures UVC is most commonly used.

### 4.1. Neonatal Phototherapy

Some newborns produce such high levels of bilirubin in the blood (hyperbilirubinemia) that it must be treated to avoid toxic reactions that can lead to severe brain damage. In the 1950s it was discovered that children who resided in daylight or sunlight at neonatal departments showed less yellowing of the skin. In the 1960s methods of phototherapy were developed as a treatment to reduce high levels of bilirubin by transforming it into water-soluble, non-neurotoxic so-called bilirubinisomerer [56].

Research has shown that the treatment efficiency is greatest with the use of blue-green wavelength range (400–520 nm with a peak at 460 nm) [57]. In Sweden approximately 5% of all new born children are treated with phototherapy [58].

There are several different phototherapy instruments on the market today. The newest equipment uses LED lighting that emits blue light to an optical fiber, which is built into a soft plastic mattress that is placed under the child. Other equipment uses fluorescent lamps that emit blue light. Incubators or luminaires with metal halide lamps or filtered halogen lamps with optical fibers are also used. A review of twelve common phototherapy devices [59] showed that the spectral distribution differs considerably between different light sources (LED, metal halide and fluorescent lamps). Generally, the LED lighting produces high spectral irradiance in the blue wavelength range (peak value around 460 nm), whereas the wavelength of fluorescent tubes can vary considerably, with a peak around 420 nm or with a widespread spectrum.

Pinto, et al. [59] measured the spectral irradiance at a distance of 20 cm from the middle of the luminaire and found a very great variability (0.2 to 8600 W/m^2^sr). Therefore, the effectiveness of treatment could differ significantly between the different types of phototherapy light fixtures and the distance between the luminaires and the newborns. Appropriate knowledge about the specific technique/luminaire which is available is also suggested by [1]. Halogen lamps produces more heat than LEDs. This is also a factor to consider in the treatment of newborns. Continuous temperature registration may be necessary, especially for halogen lamps, to ensure that the environment is safe for the baby [60].

### 4.2. Other Types of Phototherapy

Other types of phototherapy are also used in medical health care. For the treatment of acne vulgaris, acid-mediated photodynamic therapy uses visible light, wavelength 400–600 nm, together with an acidic gel to treat acne vulgaris. This method is used in Sweden at dermatological clinics, and we found a couple of devices in the inventory. According to [61,62], reactions such as stinging and erythema have been reported.

### 4.3. Dental Curing Lamp

The use of direct, light-cured, resin-based composite materials have increased rapidly during the last decades in Sweden and in other countries, and this has almost completely replaced amalgam in dental practice [63]. By applying visible light, often in the blue region, with a certain radiant energy and wavelength, the composite material quickly polymerizes and hardens. The effect can be achieved with different light sources: tungsten halogen, plasma arc curing (PAC), laser and light-emitting diodes (LED). Based on our survey among the three different county councils in Sweden, LED is dominating the Swedish market, but there are still halogen lamps available.

There are many reports showing that the irradiance from commercially available Light Curing Units (LCU), derived from the manufacture’s reports, varies greatly with respect to wavelength and irradiance, e.g., see [64,65,66] where irradiance from 0.1 to 20 kW/m^2^ with a wavelength spectrum between 380 to 510 nm are found. With the introduction of LED LCUs it has been less common with wavelengths within in the UV range, but halogen tungsten LCU can have part of its spectrum within the UVA range. There are also some direct resin-based composite materials with absorption peaks in the UVA range [67] that demand LCUs with enough intensity within the UVA range.

Many modern LCUs offer a range of modes where the irradiance, and sometimes the wavelength, can be modified by choosing different modes from the LCU. The irradiance is often lowered by pulsing the light in various frequencies or by alternating between high and low power, and there are also modes that will ramp up the irradiance in a distinct step up to a certain level. Manufactures offer different techniques to modify the irradiance [64]. The possibility to vary the wavelength will make it possible to use a wider range of photo initiators. Previously, halogen LCUs, which have a wider wavelength range (including UVA), worked with a wide range of materials. The introduction of LEDs that often have a narrower spectrum (often without any UVA), has demanded that the operator has knowledge about the spectrum of the specific LCU and the specific resin composite.

The LCU is handheld and the light is applied through a rather small opening directly to the composite material in the tooth. The crucial parameters for correct polymerization of the composite are the radiant energy expressed as J/m^2^, and that the light spectrum is matched to the optimal wavelength for the specific composite. The radiant exposure needed for commercially available composite can vary between approximately 6 J/m^2^ to 48 J/m^2^ [63,68]. Other factors that affect the curing quality are, e.g., the distance between the tooth and the active tip of the LED, the size of the area/volume to be cured, the volume of the composite material in the filling, and the size of the active tip of the LED. It is therefore impossible to state a specific curing time to achieve a high-quality curing of the composite. The operator needs to have good knowledge about these factors, including the characteristics for the LCU being used and the absorption peak of the composite material.

The intensity of the curing light has increased during recent years to increase the efficiency and to decrease the total curing time. McCusker, et al. [65] measured the “weighted irradiance” (probably the E_ν_ weighted by the L(λ)), and the maximal exposure time (t_max_) during direct exposure at different distances. A comparison with the former ICNIRP guidelines for incoherent light [69] showed that depending on the LCU used, the calculated t_max_ at 10 cm distance (which is comparable with the distance between the tip of the LCU and the eyes of the patient) varied between 0.2 min and 12 min. At 30 cm (comparable to the distance to the operator) t_max_ between 22 min to 120 min was calculated. During normal operation, direct exposure to the patient’s or operator’s eyes are not common.

McCusker, et al. [65] also measured the reflected light by introducing brackets of both metallic and composite material and measured the reflected irradiance at a distance of 10 cm. The results showed that the t_max_ for the most reflecting bracket was 62 min. Labrie, et al. [70], however, showed that for palatal position of the LCU (the LCU is placed under the front teeth with the light in the direction of the operator’s eyes) for some of the more powerful LCUs, the t_max_ could be as low as 6 s at a distance of 30 cm based on blue light hazard and the ICNIRP [69] guidelines.

The dental light-curing procedure is a handicraft. The operator needs experience in the technique and knowledge about factors such as choice of light unit and curing time to achieve high quality curing and to reduce risks. Santini and Turner [71] showed in a questionnaire study that in the UK, the knowledge of resin-based composite material and the light curing technique is poor. A large fraction of the respondents, 112/181, did not know about the features on their LCUs, e.g., low versus high intensity, the spectrum characteristics, and this indicates poor knowledge in the field. There also seems to be a confusion about whether the LCU emits UVA or only visible light. [67]. Many reports recommend the dentist to use eye protection to avoid blue light hazards during a working day and advise the dentist not to just look away from the light [63,65,67,72]. Bruzell [67] also stresses the need to choose the protective eyewear carefully since the transmission can vary greatly (0.0001–20%).

### 4.4. Disinfection Techniques Using Blue Light.

For many years UV light has been used for disinfection purposes in health care, but during recent years visible violet-blue light in the range of 405 nm has been tested as a mean of disinfecting air and surfaces and hospital rooms [73,74]. The technique is called high-intensity narrow-spectrum (HINS) light, and targets intracellular porphyrins, which are photosensitizers that absorbs 390–425 nm light and produce reactive oxygen species. The efficacy is lower than UVC light, but it can be used in areas occupied by patients. In one study, continuous HINS light showed a 27 to 75% reduction in surface contamination by staphylococci compared with control areas [75]. The authors also state that the level of exposure from this type of equipment is safe for humans and is in line with the requirements, i.e., ICNIRP [76], due to the rather low effect and large area of exposure. However, we have not found any published literature where compliance with the present guidelines has been reported.

### 4.5. Basic Types of Lasers in Medicine

There are basically seven different types of lasers used in healthcare, namely solid-state lasers, diode lasers, CO_2_ lasers, argon and krypton ion lasers, excimer lasers, dye lasers, and free electron lasers [77]. The solid-state ruby laser emits red light (λ ≈ 694 nm) and common applications are for the removal of tattoos and birthmarks. The neodymium-based Nd: YAG (host crystals of yttrium aluminium garnet) solid state laser (λ = 1064 nm) is widely used in urology, pulmonology, and gastroenterology [78,79,80]. The erbium-ion based Er:YAG solid-state laser (λ ≈ 3 μm) with pulse energies between 10 and 3000 mJ, duration of 100 μs to 1 ms, and repetition rates of about 50 p/s, is found in dentistry since it can work as a dental drill. The holmium-based solid state laser (Ho:YAG) (2.1 μm, pulse energies between 0.2 and 3 J, maximum repetition rates up to about 30 Hz, and average power up to 45 W) is used for incisions in cartilage and bone, endoscopic and open ablation of tissue, arthroscopic or percutaneous orthopedics, recanalization of vessels, and for lumbar laser disc decompression [78,81]. Diode lasers (630–980 nm) with an output power range up to 1 W are frequently employed as diagnostic or therapeutic instruments, or as positioning tools for other medical devices (e.g., MR, CT) [77,82]. Diode lasers are used to illuminate structures in biological tissues and determine the speed of moving particles (e.g., erythrocytes), for fluorescence diagnostics and for photodynamic therapy (PDT). Various tissue reactions can be induced, such as hyperthermia, coagulation, and vaporization. The carbon dioxide (CO_2_) laser (9–11 μm) is suitable for surgical applications, involving the cutting and vaporization of tissue [77]. Argon and krypton ion lasers emit light between 250 and 530 nm and 350 to 800 nm, respectively [83]. Their output power is between 5 and 10 W and most applications are found in ophthalmology, dermatology, and photodynamic therapy (PDT). The excimer laser is a pulsed gas laser emitting in the UV wavelength range from 157 to 351 nm [84] commonly used within photorefractive keratectomy (PRK), laser in situ keratomileusis (LASIK) in ophthalmology, and laser angioplasty. Unlike most other lasers, dye lasers offer the possibility of shifting the output wavelength [85,86]. The wavelength range for one dye is 50–100 nm, and it is possible to cover the range from 400 to 900 nm by employing presently available dyes. Typical applications in medicine are in laser lithotripsy and dermatology. A free electron laser (FEL) generates tunable radiation with wavelengths ranging from microwaves over visible and ultraviolet light up to x-rays. FEL is used as a surgical tool in ophthalmology (corneal tissue), otolaryngology, and neurosurgery (e.g., tumor ablation), or generalized wound healing via photo vasodilation [77]. Other areas of application are in medical research, such as spectroscopy studies of biological micro molecules, inactivation of pathogenic microorganisms and development of methods for the use of optical coherence tomography (OCT) imaging in diagnostic applications [87].

### 4.6. Laser Therapy

An application of interest is laser-induced thermal therapy (LITT), which is an emerging technique to treat primary and metastatic tumors in the brain, liver or elsewhere, where they are hard to reach with conventional surgery [88,89,90,91,92,93,94]. According to Rahmathulla, et al. [92] two main types of lasers are used, i.e., the continuous-wave Nd:YAG laser or the diode laser with wavelengths of 1064 nm and 800–980 nm, respectively. Typical laser output levels are between 6 to 15 W [89,95]. LITT is performed by implanting a laser catheter, quite often guided by other means (e.g., with ultrasound or real-time MRI), into the tumor and heating it to temperatures high enough to kill it. The catheter is implanted using advanced computer imaging techniques. The laser is guided through the catheter and allows the surgeons to limit thermal energy delivery only to the tumor. Most patients can go home the day after treatment and can quickly return to normal activities. LITT therapy is minimally invasive. It typically requires only a 2-mm incision and takes only a few minutes to perform. LITT can also help patients who do not respond to stereotactic radiosurgery or have radiation necrosis (tissue death caused by radiation treatment).

Photodynamic therapy (PDT), sometimes called photo-chemotherapy, is a form of phototherapy involving light (such as laser) and a photosensitizing chemical substance, used in conjunction with oxygen to elicit cell death (photo toxicity). For an extensive review, see Jamil and Berlien [96]. A photosensitizer drug is injected that gets concentrated only in tumor cells a few hours after injection. When the laser beam is directed toward the tumor area (cancer) it causes selective death of the cancer cells with minimal damage to the surrounding normal tissues. In addition, PDT has the ability to kill microbial cells, including bacteria, fungi and viruses [97]. It is used clinically to treat a wide range of medical conditions such as macular degeneration, psoriasis, atherosclerosis, acne, cancers (e.g., lung, bladder, prostate, and skin) and has also shown some efficacy in anti-viral treatments, including herpes [96,97,98,99]. It is recognized as a treatment strategy that is both minimally invasive and minimally toxic. Photosensitizers have been employed to sterilize blood plasma and water to remove blood-borne viruses and microbes. It has also been considered for agricultural uses, including herbicides and insecticides. PDT reduces the need for delicate surgery and lengthy recuperation, and there is minimal formation of scar tissue and disfigurement.

### 4.7. Laser Surgery

During surgery, laser is used to cut tissue instead of using an ordinary scalpel. The laser beam vaporizes soft tissue with high water content. Typical surgical lasers include CO_2_, erbium, dye, argon, and Nd:YAG lasers. In dermatology and plastic surgery lasers are used to treat various skin conditions such as scars, vascular and pigmented lesions. Laser surgery is commonly used in ophthalmology for treatment of refractive errors as well as non-refractive conditions. For example, LASIK is used for correction of near and far-sightedness in vision, photorefractive keratectomy (PRK, LASEK) is used to reshape the cornea without cutting a flap with a scalpel, and laser thermal keratoplasty improves near vision by placing a ring of concentric burns in the cornea. Examples for treatment of non-refractive conditions are phototherapeutic keratectomy (PTK) in which opacities and surface irregularities are removed from the cornea, and laser coagulation to cauterize blood vessels in the eye to treat various conditions. Other medical areas where laser application is found useful is in foot and ankle surgery, oral and dental surgery, gynecology, genitourinary, general and thoracic surgery, otorhinolaryngology, orthopedic, neurosurgery and gastro-intestinal medicine. For a more extensive review of laser applications in surgery, see [100,101,102,103,104,105,106].

### 4.8. Laser Diagnostics

The characteristics of laser light are well suited for non-invasive exploration of tissue structure and high-resolution imaging for diagnostic purposes. Among methods used for diagnostic purposes are optical coherence tomography (OCT), auto-fluorescence bronchoscopy (AFB), fibred confocal fluorescence microscopy (FCFM), fluorescence lifetime imaging microscopy (FLIM), diffuse reflectance, Raman spectroscopy, optical molecular imaging (OMI), optical imaging, laser Doppler flowmetry (LDF) and laser Doppler perfusion imaging (LDI). Some of these applications are briefly summarized and referenced below.

Optical coherence tomography (OCT) is an established medical imaging technique that typically uses NIR light to capture micrometer resolution three-dimensional images from e.g., biological tissue. OCT is based on low-coherence interferometry and the use of long wavelength light allows it to penetrate into the scattering medium. Confocal microscopy, which is another optical technique, typically penetrates less deeply into the sample but with higher resolution. Depending on the light source (e.g., super luminescent diodes, ultra-short pulsed lasers, and super continuum lasers) OCT has achieved sub-micrometer resolution with sources emitting over a ~100 nm wavelength range. OCT is used across several medical specialties including ophthalmology, cardiology, dermatology, rheumatology, and oncology is also widely used in medical research [107,108,109,110,111,112,113].

The optical molecular imaging (OMI) concept is based on optical imaging and diagnosis of pathologic tissue changes. Such imaging and diagnosis is focused on observing and visualizing tissue areas in the molecular range by means of disease-specific dyes. The laser light source must have a wavelength within the NIR spectral range between 650 and 1200 nm. Optical imaging offers various medical applications including for instance the detection and evaluation of superficial dermatome, squamous cell carcinoma, tumors of the base of the tongue, hyperplasia, dysplasia, bronchial carcinoma and malignant glioma [114,115,116,117]. Optical imaging is also used for the diagnosis and monitoring of inflammatory rheumatoid diseases or intra-operative monitoring of cardiac ischemia [118,119].

Fluorescence-lifetime imaging microscopy (FLIM) is an imaging technique for producing an image based on the differences in the exponential decay rate of the fluorescence from a fluorescent sample [108,120,121,122]. The lifetime of the fluorophore signal, rather than its intensity, is thus used to create the image. FLIM can be used in confocal microscopy, two-photon excitation microscopy, and multiphoton tomography. The method uses a light source that is pulsed or modulated at high frequency (up to 500 MHz) such as an LED, diode laser or a continuous wave source. FLIM is primarily used as a method to detect photosensitizers in cells and tumors, in clinical multiphoton tomography to detect intradermal cancer cells as well as pharmaceutical and cosmetic compounds. FLIM imaging is particularly useful in neurons, where light scattering by brain tissue is problematic for ratio metric imaging. Laser florescence imaging allow the early detection and quantification of initial caries formed around orthodontic brackets minimizing the damage of caries lesions in orthodontic patients [123].

Raman spectroscopy is used to observe vibrational, rotational, and other low-frequency modes in a system. The method relies on inelastic scattering (Raman scattering) of monochromatic laser light emitted in the visible near infrared, or near ultraviolet range. Raman spectroscopy has a wide variety of applications in biology and medicine. It has for instance been used to confirm the existence of low-frequency phonons in proteins and DNA and used as a non-invasive technique for real-time, in situ biochemical characterization of wounds [108]. Raman signals are however weak why imaging requires high laser power (typically > 10 mW), very sensitive detectors and long data acquisition times (>30 min). This imaging technique is thus unsuitable for clinical applications [108].

Laser Doppler flowmetry (LDF) and laser Doppler perfusion imaging (LDI) are methods that use the Doppler shift in a laser beam to measure the velocity in transparent or semi-transparent fluid flows or the linear or vibratory motion of a reflecting surface. Beams of collimated, monochromatic, and coherent laser light with wavelengths in the visible spectrum (λ 390–750 nm) are used to ensure coherence. Typically, He-Ne, Argon ion, or diode lasers are used. LDF/LDI is used as a technique to partially quantify blood flow in human tissues such as skin. The beam from a low-power laser (usually a laser diode) penetrates the skin sufficiently to be scattered with a Doppler shift by the red blood cells and return to be concentrated on a detector. These measurements are useful to monitor the effect of exercise, drug treatments, environmental, or physical manipulations on targeted micro-sized vascular areas. The technique is also being used in clinical otology for the measurement of tympanic membrane (eardrum), malleus (hammer), and prosthesis head displacement in response to sound inputs of 80 to 100 dB. It also has potential use in the operating room to perform measurements of prosthesis and stapes (stirrup) displacement. Important clinical application is also found in dermatology since malignant skin tumors have higher perfusion than benign nevus and basal cell carcinomas. When measuring the blood perfusion there is a possibility to differentiate between various types of skin tumors.

Endothelial dysfunction is one of the key events in the development of atherosclerosis and has been confirmed in patients with cardiovascular related diseases. Owing to its accessibility, the skin microcirculation is frequently used as a model to assess the general condition of the endothelium. Blood perfusion imaging (i.e., LDF, LDI), in combination with iontophoresis, post-occlusive reactive hyperemia or thermal challenge, has been proven to be an excellent tool for endothelial function studies [124]. Another important clinical application is the assessment of the skin blood flow response to a provocation in diabetic patients. Already in its early stages, the diabetic disease impairs the sympathetic nervous system, which controls skin blood flow. Stimulating the microcirculation, either by a drug or cold exposure, and then measuring the vascular response with LDF, LDI, LASCA or similar technology allows for a quantification of the sympathetic control function [125,126].

In addition, measuring perfusion in wound healing is useful for several disciplines, such as diabetes care, surgery and geriatrics medicine. Infections and inflammation of the wound increases the perfusion that is easily detected with laser Doppler technology. For instance, leg ulcers and wounds can be monitored easily and without physical contact, which is a benefit regarding contamination and discomfort issues. Burn wounds are not always easy to judge clinically. Early assessment of burn depth is crucial to avoid unnecessary surgery or potential hypertrophic scarring. The status of the skin microcirculation reflects the burn depth and changes in the skin blood flow over time will reveal the wound healing potential. Increased activity indicates that the microcirculation is functioning and that there is a higher degree of wound healing potential [127].

Laser Doppler imaging (LDI/LDF) or laser speckle contrast analysis (LASCA) is used to follow vascular changes in order to monitor or understand the underlying mechanisms. These applications use laser radiation within the waveband of about 630–785 nm. One example of application is to diagnose or monitor disturbances in finger microcirculation due to Raynaud’s syndrome. The phenomenon is characterized by a vasospasm in the extremities as a response to cold temperatures or other sympathetic stressors, for example noise. LDF, LDI and LASCA has also proven useful to distinguish between secondary and primary Raynaud, as well as differentiating between established and early disease [128,129]. In orthodontics, LDF has found its application in assessing the vitality of the tooth during or prior to undergoing orthodontic treatment [123].

### 4.9. Biological Effects of Laser Radiation

The scientific literature that addresses the biological effects of exposure to optical radiation such as lasers is very comprehensive. The reports published by the European Commission and ICNIRP [1,130,131,132] provide extensive and very good overviews of the current state-of-the-art regarding biological effects and other aspects of laser radiation.

Adverse health effects of laser radiation are the result of one or more biophysical interaction mechanisms (i.e., photochemical, thermal, thermo-acoustic) that vary depending on wavelength and exposure duration. It is clear that an exposure to laser radiation may cause adverse health effects across the entire optical spectrum from ultraviolet (λ > 180 nm) in to the far infrared (λ < 10^3^ µm). The injury threshold varies greatly across the optical spectrum due to biological and structural differences of tissues and organs that are potentially at risk. Skin and eyes are the main target for health effects, and wavelength plays an important role regarding which part of the skin or eye that absorbs the radiation most and which type of interactions are involved. Exposure to short wavelength ultraviolet laser radiation is most important for photochemical effects whereas thermal effects are most dominating in the infrared region. Some examples of photochemical effects are erythema (reddening of the skin), conjunctivitis, photo keratitis (corneal inflammation) and cataract. The primary effect of visible and near infrared laser radiation is damage to the retina. The retina is very susceptible to radiation in the region (i.e., λ = 400–1400 nm) because of the transparency of the ocular media and the focusing properties of the eye. In the mid- and far-infrared part of the optical spectra (i.e., λ > 1.4 µm) laser radiation primarily damages the cornea.

The biological effects of laser radiation can broadly be divided into acute and chronic. In general, acute effects will only occur if the exposure exceeds a threshold level. This critical exposure level usually varies among individuals. Most exposure limits are based on studies of thresholds for acute effects and derived from statistical consideration of these thresholds. Therefore, exceeding an exposure limit will not necessarily result in a health effect. The risk for getting an adverse health effect will increase as exposure levels increase above the exposure limit defined in the EU Directive [133]. However, persons who are abnormally photosensitive may suffer adverse effects at levels below the exposure limits. In general, the long-term effects of repeated and chronic exposures to laser radiation in general are not well understood. Chronic effects often do not have a threshold below which they will not occur. Risks for adverse health effects may thus be reduced through lower daily exposure levels.

### 4.10. Safety Regulations and Guidelines

ICNIRP has published guidelines on limits of exposure to laser radiation that apply to wavelengths from 180 nm to 1 mm and to exposure durations between 100 fs and 30 ks (about 8 h) [131]. The purpose of these guidelines is to establish the maximum levels of exposure to laser radiation that are not expected to cause adverse biological effects to the eyes and skin. The guidelines apply to all human exposure to optical radiation emitted by lasers. The exposure limits for lasers were derived on the basis of a robust set of experimental damage threshold data, which describe the dose-response relationships for the biological effects of laser radiation on the eye and skin. These damage-threshold doses depend on the wavelength, exposure duration and spot size. Presented exposure threshold limits should be used as guidelines for controlling human exposure to laser radiation.

These guidelines are considered to be adequate for the general population as well as for occupational exposure. However, the threshold limits should not be regarded as sharp demarcations between ‘‘safe’’ and ‘‘dangerous’’ exposure levels. Exposure at levels below the exposure limits should not result in adverse health effects. The limits incorporate the collective knowledge generated worldwide by scientific research and experience of laser safety, and these limits are based upon the best available published information. The most effective method to control laser hazards to the eye and skin is totally enclose of the laser and all beam paths. For conditions where this is not possible, partial beam enclosure, laser eye protectors, restricted access to beam paths, and administrative controls may be necessary.

The widespread and increasing use of lasers in medicine should be considered as a potential safety problem for staff and patients [1]. Most laser applications for diagnostic purposes are of Class 1 (<0.4 mW) and Class 2 (<1 mW) and thus considered harmless for the eye and skin. Laser applications for therapy and surgery include equipment with higher output power, i.e., Class 3B (<5 mW), 3R (5–500 mW), and 4 (>500 mW) which are associated with a “Low risk to eyes/No risk to skin”, “Medium to high risk to eyes/Low risk to skin”, and “High risk to eyes and skin”, respectively. Issues such as calibration, maintenance and practical handling of the equipment are of great importance in this context. A competent and efficient safety organization and routines must be in place in every health care organization for avoiding negative health effects of laser radiation on patients as well as on operators. Likewise, protection must be given more attention and personnel involved in all aspect of the use of laser equipment for medical purposes should be well-trained and updated on safety issues. For further information regarding different aspects on laser safety, see the comprehensive overview authored by Henderson and Schulmeister [134].

### 4.11. Medical UV Phototherapy Treatment

Skin diseases such as psoriasis and eczema can be treated with UV radiation. This uses either narrowband UVB radiation where the lamps emit UV light with the main peak at 310 nm or UVA combined with tablets (psoralens) which is called PUVA treatment. For some eczema treatments combinations of UVB and UVA are used.

The most common types of lamps used are TL01 and PUVA lamps. Both full body and partial body (hand/feet) phototherapy units are used depending on the diagnosis. The full body standing units are covered with fluorescent tubes and during treatment the patient will be positioned inside the unit. Partial body units are commonly adjustable smaller units wherein the patient places their hands or feet. The unit is commonly covered with cloth to avoid radiation to the surroundings.

The dermatologist ordinates a treatment schedule were the initial dose is defined depending on the diagnosis and the sensitivity of the patient (other diseases, skin type, previous UV exposures etc). The treatment dose is then often increased in predefined steps, during the treatment period to achieve highest possible treatment effect and to avoid unwanted side effects such as erythema and burns. The Swedish National Board of Health and Welfare has an ongoing work on a national recommendation for psoriasis treatment and the work is expected to be finished in 2019 (www.socialstyrelsen.se).

The treatment dose is expressed in J/m^2^. Depending on the manufacturer the adjustable treatment dose is expressed as J/cm^2^, or as a treatment time (minutes, seconds).

For each treatment a nurse sets the dose, but at some clinics the patients themselves set the dose. Some newer UV treatment units provides automatic dose adjustments, were each patient’s treatment schedule is pre-programmed and the patient just enter their personal code number at each treatment.

The dominating fluorescent tubes used in medical health care in Sweden are PUVA and TL01, where PUVA emit broadband UVA light and TL01 emit narrow-band UVB (311nm).

#### Potential Risks during UV Treatments

Acute potential risks after UV treatment are damage to the skin and eyes and this has also been reported after UV therapy [135,136,137]. This is also regulated by the Swedish Work Environment Authority for occupational exposure [138,139] For patient exposure some paragraphs are included in the SSM regulation of sunbeds [140]. The acute health effects that might occur after exposure to UV is skin erythema, or if the exposure is very high; burns with blistering and peeling. The damage occurs first a few hours after irradiation. For the patient, the dermatologist prescribing a specific individual starting dose and a treatment schedule where the treatment does increases in small steps reduces this risk. There is sufficient evidence that long-term exposure UV radiation (UVA, UVB and UVC) causes cancer of the skin, especially malignant melanoma and basal cell carcinoma and UV radiation has therefor been classified as carcinogenic by IARC [141]. PUVA treatment has been classified as carcinogenic by IARC [141], while UVB treatment alone has a weak association to cancer based on epidemiological studies. However, when animal and biological studies also where included, IARC concluded that there were sufficient evidence that UVB alone are carcinogenic.

Interesting noting is that the Medical Products Agency in Sweden recommendation for Psoriasis treatment [142] indicates that broad band UVB treatments are considered to be a very safe form of treatment with respect to increased risk of skin cancer, but that animal studies have shown an increased risk of narrow band UVB compared to broad band UVB. They also state that this has not been confirmed among patients treated with narrow band UVB. The Medical Products Agency also concludes that oral intake of psoralen in combination with UVA treatment is associated with an increased risk of squamous cell carcinoma. They also state that this has not been shown for topical PUVA.

We have previously measured the irradiance in human whole-body UV therapy units (unpublished data) and compared those to the hygienic guidelines to UV radiation [140] for the unprotected skin. The maximum exposure time for TL01 fluorescent tubes was calculated to 20 s. For PUVA fluorescent corresponding figure was about 20 min.

Since the exposure level inside the UV unit is extremely high, especially when using the TL01 (UVB) lamps, personnel should never be inside the units with any parts of their body without protective clothing to avoid overexposure [138,139]. The measurement procedures to confirm the irradiance, which is also a demand in SSMFS [140] are commonly done with the technician standing inside the full body unit with protective clothing and protective eyewear holding a measurement probe in the hands. Some manufacturers recommend this method. This procedure could potentially be risky since a small opening in the clothing could lead to severe effects on the skin or eyes. At VCC a new method has been developed where the instrument is placed on a tripod inside the full body unit. The measured value is then adjusted to what should have been achieved in a real situation with a person placed inside the unit using an empirical evaluated correction factor.

Potential risks during UV treatment are for instance that the treatment dose is by mistake too high, this is especially important if patients are entering the dose themselves. Other potential risks are that the personnel are exposed to UV, either by mistake during measurement procedures or for instance when helping children during especially hand/feet treatments if protective clothes are not used.

### 4.12. UV Disinfection

UV and especially UVC are used to disinfect equipment for instance by using UV cabinets where in the equipment is placed. These are closed processes where UV light is omitted to enter the protective barriers to avoid accidental exposure to the personnel, for instance, by using automatic switch-off irradiated UV light when the door is opened.

There are also open disinfecting techniques, like Ultraviolet germicidal irradiation techniques (UVGI), which disinfect the air in for instance health facilities. Upper room installation is used to clean the upper layer of air and in combination with ventilation this could be an effective technique to control airborne infectious particles [143,144]. During normal operation the UVC light is used in upper room installation and the irradiance levels are not in conflict with present guidelines [144]. There are also open UVC cleaning system that irradiates a larger part of a room and not only the upper layer [145,146]. Using these devices need special precautions since the exposure level within the room will be above ICNIRP guidelines [147] during irradiation. People should not be inside the room during disinfection, but accidents have been reported. This technique is available in Sweden today and is to our knowledge in limited used at some hospitals. There are also other types of UVC emitting cleaning devices on the market [148,149] but to our knowledge the usage of those is limited in Sweden today and the technique has also been questioned with respect to cost efficiency and occupational safety [150,151]. In a recent report from SCHEER [152] UVC lamps are discussed extensively and they conclude that adverse effects to human skin and eyes could be caused by accidental exposure to UVC lamps. This is also regulated in Sweden [138,139]. In line with IARC [141], SCHEER [152] also states that UVC radiation can cause cancer, but that quantitative cancer risk from exposure to UVC lamps cannot be obtained due to lack of data.

Since the clinics that are using open UVC systems do not have any former experience of UV light and potential risks it is of importance that the introduction of this technique is done carefully and that the personnel get proper education on the use, the routines and safety when using open UVC systems.

### 4.13. General Comments on the Use of Optical Radiation in Health Care

The European Parliament and the Council of the European Union has adopted a directive that lays down minimum requirements for the protection of workers from risks to their health and safety arising or likely to arise from exposure to artificial optical radiation during their work [133]. The Directive refers to adverse effects from exposure to the eyes and skin. Adherence to the exposure limits, as set out in Appendix II of the Directive, should provide a high level of protection from side effects that may result from exposure to laser radiation. The Directive also introduces measures protecting workers from the risks associated with optical radiation to the eyes and to the skin. These measures are intended not only to ensure the health and safety of each worker on an individual basis, but also to create a minimum basis of protection for all workers to avoid possible distortions of competition. The corresponding Swedish provision for workers’ exposure to artificial optical radiation is solely based on the EC Directive 2006/25 [138,139]. The Commission has also published a non-binding guide to good practice for implementing the Directive 2006/25/EC in order to help employers to better understand the technical provisions of this Directive [130].

Light treatments in health care are widespread and the dominating techniques are dental curing lamps and neonatal phototherapy. Both techniques have been improved and the usage of LED light has increased. Since LED does not emit UV light the safety improvements are obvious compared to the older halogen technique. But the blue light hazard still needs to be taken seriously and ICNIRP stated in a workshop on non-ionizing radiation in health care that the widespread use of optical radiation in health care suggest that there could be potential safety issues and stressed the importance of staff training, maintenances and calibration and also mentioned protective eye wear as of special importance [7].

The treatment efficiency using LED or halogen lamps for neonatal treatments needs special attention and the knowledge of this at neonatal centers would be of interest to study.

The UV phototherapy units used in health care do, especially for the narrow spectrum UVB (TL01) treatments, produce an effective irradiance that are above the Swedish regulations. It is therefore of importance to have good knowledge of the safety aspects of UV and that the treatment dose is adjusted carefully for each patient and that the irradiance in each unit is checked regularly. Personnel should not be exposed above the limit values and care should therefore be taken during the measurement process as well as during treatment of patients.

Open UVC light used for disinfection in for instance operation theatres or patient rooms have been introduced on the Swedish market and overexposure to personnel can be avoided by carefully using the equipment in line with the manufacturer’s instruction.

It would be of interest to study how the safety work at dermatological clinics are organized and how the treatment doses are set and administrated. The knowledge and safety routines among the personnel at other clinics using open UVC light for disinfection purposes would also be of interest to study.

Lasers are used for diagnostic or therapeutic purposes within many medical disciplines such as in ophthalmology, dermatology, oncology, odontology, and clinical physiology. Laser radiation is predominantly absorbed in the outer layers of the body why skin and eyes are the main targets for acute or chronic biological effects. The daily individual exposure dosage for an individual user is not easy to determine. Risk assessment based on exposure threshold limits presented in current provisions and guidelines is consequently difficult to do. The use of laser applications in medicine should be considered a potential safety problem for staff and patients. Safety and protection issues must thus be given more attention to avoid negative health effects due to laser radiation on patients as well as the work force. Personnel involved in all aspects of the use of laser equipment for medical purposes should be well-trained and updated on safety issues.

Based on the result of this review the following research and development needs are identified for medical application using optical radiation in Swedish health care;

- An in-depth risk and safety analysis of exposures for patients and staff for applications such as class 3 or 4 laser products, UV therapy units and dental curing light.
- A better understanding of long-term effects of repeated and chronic exposures to optical laser radiation.
- Overview of current safety organization and safety routines at our Swedish hospitals.
- Inventory of the use of different types of light therapy units for treatments of neonatal phototherapy and the knowledge about the radiation spectrum and its efficiency of treatment outcome.

## 5. Discussion

NIR is widely used for various applications in the health care sector. We have identified the following applications where the exposure levels are high and acute effects for patients are possible: TMS, MRI, ESU, UV treatments, laser therapy and harmonic scalpels in surgery. There are significant risks if the equipment is not used properly. When the exposure limits for occupational exposure have been exceeded for TMS, UV treatments, electrosurgical diathermia, laser therapy, adverse effects have been reported, e.g., burns on the patient’s hands/body due to excessive exposure during UV treatment, ferromagnetic objects get stuck in the MRI bore and burns in patient during electrosurgical procedures. However, there are probably more unrecorded accidents or deviations with NIR than with ionizing radiation, where there are well functioning routines and requirements to produce accident reports.

There are no clear demands from authorities on accident reports when using NIR. This is supported by Sienkiewicz, et al. [1] where the authors also discuss the generally low reporting of NIR-associated deviations, e.g., ICNIRP and SCENIHR for MRI. There is also a lack of knowledge of combined exposure with other agents, since it has become more common with hybrid techniques, where combinations of techniques occur, e.g., ionizing radiation and magnetic fields are used (PET-MRI) for diagnostics [4,132]. We have not covered combinations explicitly in this report.

To be able to control the exposure to the patient or staff during treatment, information is needed on the exposure level that is used and how it can be adjusted. NIR is a complex area where the general knowledge about exposure characteristics in health care is poor. The regulations for optical radiation and EMF are complex with frequency and time dependence that is not easy to interpret. When performing measurements, good knowledge about frequency sensitivity of the instrumentation and source calibration is needed. These aspects are an almost impossible task for a clinic and a biomedical engineering unit to handle today. Therefore, there is a need for practical guidance for specific applications both with respect to demands from the authorities, but also how to succeed with the safety work within the area.

In general, occupational exposure is covered by national legislation, but it is questionable if these regulations are fully implemented in the health care sector. Medical use is covered, to some extent, by national legislation where requirements such as exposure control (UV) and the practitioner’s profession (laser, UV) are defined.

It has not been possible within the scope of this project to count or even estimate the number of procedures that are done yearly for the different techniques. We have tried to extrapolate a figure for MRI, but for other applications the task is impossible. This is partly due to a lack of continued reporting from the individual clinics. To achieve any reasonable estimation of NIR use, specific clinics/hospitals would have to log all procedures performed within a specific category during a short, well-defined time interval. The same method could be used to record deviations or accidents from a specified medical procedure.

Throughout our work we have noticed that it is hard or nearly impossible to map all different applications—even within a single hospital. Compared to the use of ionizing radiation in health care, which is carefully organized with an authorization procedure for each piece of equipment used, the use of NIR in general does not have a dedicated organization. Our experience is that the quality of safety work differs between clinics and that dedicated, reliable information about safety during use of NIR in medical procedures is sparse. To improve the knowledge in safety and NIR in the health care sector, there is a need for clear, evidence-based information from reliable sources, and it should be obvious to the user which source has the appropriate information.

The knowledge of treatment procedures and safety aspects is commonly held by, and passed on from, manufacturers, colleagues, medical physicists and biomedical engineers at the hospital. The knowledge regarding these issues is up to the clinic and the biomedical engineers assigned to the clinics. At each hospital, a medical service and maintenance organization is available to support medical equipment including applications that use NIR. Their basic knowledge about measurements of NIR is obtained from the manufacturers, and we have noticed that there are uncertainties in measurement methods and instrumentation. One way to overcome these difficulties would be to assign a medical physicist/biomedical engineer to NIR who could work as an expert in safety, measurement techniques and help with safety training of staff. This has already been introduced in MRI applications where an MR physicist, often but not always with a background as medical physicist, works as a consultant in safety issues and training of staff.

In general, there are no education programs about NIR, and risks (with the exemption of MR) to our knowledge are sparsely covered in the university programs. The medical physicist programs cover NIR but only to some extent. Demanding safety training at the clinics could be one way to increase the knowledge about NIR and its health risk. Moreover, it is also important that the training recur regularly within the clinic. The hospitals need to have knowledge about the physical aspects of NIR and they need to be able to interpret the regulations and to understand the safety aspects for each specific medical device.

We suspect that the training and education of physicians and nurses about NIR is very sparse. Lee and Lee [153] conducted a survey among Irish medical students on their knowledge of ionizing radiation safety in healthcare. Their knowledge about radiation safety was limited. For example, as much as 74% were not aware of any laws governing the prescription of radiology investigations. We think that the same can be said about the knowledge about non-ionizing radiation and the laws and guidelines in this area.

Nevertheless, to improve the knowledge in safety and NIR in the health care sector, there is a need for clear, evidence-based information from reliable sources, and it should be obvious to the user where to find that information.

## 6. Conclusions

NIR is extensively used in health care at many clinics and units for a variety of applications. The exposure to patients and staff needs to be carefully controlled to avoid acute health effects for MRI, TMS, electrosurgical diathermia, UV treatments, laser therapy, and harmonic scalpels in surgery. The knowledge about long-term effects from MRI is sparse. The knowledge is also sparse regarding possible synergistic or additive adverse health effects for hybrid equipment, e.g., when two or more NIR applications are applied simultaneously.

The putative increased use of rTMS requires analyses of the safety procedures and research of possible negative effects.

We conclude, based on our own experience in this field, that knowledge about the health effects in relation to the use of NIR in health care is poor. To achieve more precise and convincing evidence a well-designed documentation/investigation is required. This kind of study will require careful consideration with a clear research question and well-chosen study group. Systematic reviews on scientific literature on health effects from the use of NIR in health care both from a patient but also a worker perspective is also warranted.

Finally, we have also identified a lack of dedicated information on the safety aspect for the use of NIR in health care. Health care professionals need a reliable source of information from a major national authority. Today it is not obvious where to find information, and the information that is available is sparse.
